# Between government policy, clinical autonomy, and market demands: a qualitative study of the impact of the Prescribing Analysis System on behavior of physicians in South Korea

**DOI:** 10.1186/s12913-015-1059-x

**Published:** 2015-09-21

**Authors:** Dong-Sook Kim, Green Bae, Soo Yeon Yoo, Minah Kang

**Affiliations:** Review and Assessment Research Institute, Health Insurance Review and Assessment Services, 267 (Seocho-dong) Hyoyeong-ro, Seocho-gu, Seoul, 137-706 South Korea; Bioethics Policy Studies, Ewha Womans University, 314 Posco, 52 Ewhayeodae-gil, Seodaemun-gu, Seoul, 120-750 South Korea; Department of Global Health and Population, Harvard School of Public Health, 1635 Tremont Street, Boston, MA 02120 USA; Department of Public Administration, College of Social Sciences, Ewha Womans University, Seoul, 120-750 South Korea

**Keywords:** Trust in government, Prescribing Analysis System (PAS), Focus Group Interview (FGI), Prescribing behaviors, Policy implementation, Policy tools

## Abstract

**Background:**

In South Korea, the Health Insurance Review and Assessment Service manages the Prescribing Analysis System (PAS) to evaluate the appropriate use of medication. To achieve the system’s goal of changing prescribing behavior, it is critical to understand how physicians respond to the PAS. This study analyzes the opinions of South Korean physicians about the PAS, the way it is used, and factors affecting prescribing behavior.

**Methods:**

A qualitative, exploratory approach was used, with four focus groups of physicians from different specialties. A semi-structured guide was used to explore their opinions. Transcripts of the discussions were analyzed by the authors, who independently considered content using uniform categories. Common themes were extracted and used to gather results and draw conclusions.

**Results:**

Physicians acknowledged some positive aspects of the PAS but, overall, had mainly negative impressions of the system, and particularly, the evaluation reports that it generates. They reported that their prescribing behavior was affected by predisposing factors, including experiential, environmental and psychological factors. Physicians reported that their negative perceptions regarding the regulations were primarily influenced by concerns about maintaining their autonomy and expertise. However, their strong resistance to these perceived infringements on their independence may be considered inconsistent in relation to their professional autonomy as there was an equally strong concern about market competition. Physicians’ objections to the PAS are more likely to have been caused by deeply rooted distrust of the government agency in charge of the system.

**Discussion:**

Interestingly, we found that physicians’ strong resistance to perceived violations of their autonomy seems somewhat inconsistent and contradictory. While they are very positive about new information or printed materials provided by pharmaceutical representatives, they are less enthusiastic when it comes to governmental guidelines or standards. Similarly, they appear to willingly accept situations in which they believe they should comply with patients’ demands as a means of surviving in a competitive market. It is notable that physicians’ negative perceptions of PAS seemed to be aggravated by suspicion and distrust regarding the purpose of this program.

**Conclusions:**

Because of widespread beliefs in professional autonomy, market competition, and a deep-seated distrust of the system, it would be difficult for the government to persuade physicians to change their prescribing behaviors using only the PAS. Successful implementation of the PAS will not only require its improvement as a policy tool, but also the creation of a social consensus regarding the PAS.

## Background

According to the World Health Organization (WHO), rational use of drugs means that patients receive medication appropriate to their individual clinical needs in the right dosage for an adequate period of time at the lowest possible cost [1]. To improve rational drug prescribing, several countries have implemented a prescribing monitoring system. For example, the UK, Spain, and Sweden use national prescribing monitoring and feedback systems. Their prescribing indicators include the percentage of generic prescribing, cost of statin prescriptions, and antibiotic prescribing rates.

However, physicians’ behavior has not necessarily changed since the introduction of these systems. Worldwide, approximately two-thirds of all prescriptions for antibiotics prescribed by physicians are known to be for the treatment of respiratory tract infections (RTIs) [2]. According to Gulliford et al. [3] antibiotics are prescribed for 36.5 % of common colds in the UK, 48.7 % in France [4], 39.7 % in Spain [5], 16 % in Holland [2], and 7 % in Sweden [6]. When Akkerman et al. [7] surveyed 146 physicians to find determinants of antibiotic overprescribing for sinusitis, tonsillitis, and bronchitis patients, only 50 % of them reported following national guidelines, which indicate that antibiotics should not be prescribed for common colds.

Antibiotic prescription abuse is also a serious issue in South Korea. In 2000, the total antibiotic prescribing rate was 57.9 % [8]. In 2002, the antibiotic prescribing rate for the common cold was 73.33 %, and the total antibiotic prescribing rate was 42.39 % [9]. Nonsteroidal anti-inflammatory drugs and corticosteroids also have some issues related to overuse [10, 11]. As a response, in 2001, South Korea introduced the Prescribing Analysis System (PAS) to promote appropriate prescribing. It is not yet clear, however, that the program has produced sufficient changes in prescribing behavior. For example, in 2013, the average rate of antibiotic prescription for upper respiratory tract infections by private clinics was substantially higher than in tertiary hospitals (43.33/hundred visits vs. 23.99/hundred visits, respectively) [12].

In this research, we aimed to understand the impact of the PAS on physician behavior, and whether it has the potential to reduce pharmaceutical misuse or abuse and manage pharmaceutical expenditure by reducing unnecessary and inappropriate prescriptions. We attempted to identify factors affecting physicians’ prescribing patterns, examined their perceptions of the PAS, and assessed the system’s strengths and limitations. Our research questions were: 1) What factors do physicians report as affecting prescribing behavior? 2) What are physicians’ perceptions of the PAS? 3) Do physicians perceive the program to be effective? If not, what are the main reasons? and 4) Are there ways to improve the current system to make it more effective?

## Methods

### Participants

We conducted focus group interviews on May 7–14, 2009. Four medical specialty areas were identified as having the most outpatient visits and prescriptions in the Seoul and Kyungki-do areas: internal medicine, otorhinolaryngology, pediatrics and primary care. We recruited 28 physicians across the specialties and geographical areas, and 27 agreed to participate in the study. They were divided into four focus groups by specialty, each consisting of six to seven physicians. In each focus group, participants’ ages ranged from 39 to 56 with an average age of 40. There were two females and 25 males. The gender distribution of primary care physicians in South Korea is 86 % male and 14 % female. Six participants worked in the Kyungki-do area, and the rest in Seoul (Table 1).

**Table 1:** Characteristics of participants

	Total	Participants			
		Internal medicine specialist	Otorhinolaryngologist	Pediatrician	Primary care physician
		(G1)	(G2)	(G3)	(G4)
No. of participants	27	7	7	7	6
Average age (years)		49.3	42.6	46.9	46.5
Age range (years)		44–51	39–50	41–56	44–48
Gender: male/female	25/2	6/1	7/0	7/0	5/1
Location: Seoul/Kyungki-do	21/6	7/0	6/1	4/3	4/2

Unit: Number of people, ages in years

Focus group interview questions were generated by literature review and discussion among the authors of this study. Participants were asked to reflect on their prescribing behavior and their experiences and perceptions of the PAS. In each focus group, a group discussion lasting one-and-a-half hours was conducted on the following topics: 1) determinants of prescribing behavior; 2) perceptions and attitudes toward the PAS; 3) PAS indicators; 4) feedback methods used by the PAS; and 5) overall opinions and comments about the system.

Interviews were conducted at a research center with videotaping facilities. Two of the authors, who are health policy researchers and familiar with government decision-making processes, moderated the focus group discussions as facilitators. Other researchers participated in the focus group as observers.

The study was approved by the Health Insurance Review and Assessment Service’s (HIRA) Institutional Review Board. Informed consent was obtained from the participants at the beginning of each session. Pseudonyms were used in data analysis to protect participants’ anonymity.

### Coding and analysis

Focus group interviews were videotaped and transcribed verbatim by a professional transcriber. All four researchers monitored the interviews and, upon completion of each one, wrote reflective notes and memos. The researchers conducted hour-long sessions for debriefing and discussion, following the completion of each group interview.

The four resulting transcripts were coded by all four authors. We used open-coding of transcripts to identify key words, phrases, and statements. As far as possible, the codes and categories were created for consistency in reflecting emerging ideas, rather than merely describing topics. In the initial line-by-line coding process, researchers tried to observe data analytically without preconceived assumptions. Through iterative reading, we identified repeated and notable words, phrases, and patterns among the participants’ responses. Several common themes emerged from the first stage of ‘interpretive’ reading, while others emerged during, or as a result of, several additional rounds of reading. We then collapsed the codes and categorized them into themes by similarity of meaning within our research questions. Disagreements and differences in describing, synthesizing, and explaining the data were resolved by continuous discussions. Emerging codes, categories, and themes were entered into matrices by specialties to enable comparisons and contrasts within and among groups.

### Prescribing Analysis System (PAS)

The South Korean government adopted the PAS in 2001 to reduce misuse and abuse of prescription medicines, and to promote proper usage, by improving each institution's autonomous management of medication. There are indicators to measure performance in three categories, including antibiotic prescription, injection frequencies, and the medication cost per day of use, which have been tracked and recorded since 2001. Later additions include the number of items per prescription (2003), the proportion of high-priced prescriptions (2003), and the duplication rate of NSAIDs (2005) (Table 2). The PAS reports prescribing tendencies of medical care institutions in a comparative format and provides feedback to each institution.

**Table 2:** Indicators used in the Prescribing Analysis System (PAS)

Categories	Indicators
Injections	Injection prescription rate
Antibiotics	Antibiotic prescription rate (all diseases)
	Prescription rate for acute upper respiratory infections
No. of drugs per prescription	No. of drugs per prescription (for all diseases)
	No. of drugs per prescription (respiratory diseases)
	No. of drugs per prescription (musculoskeletal diseases)
	Prescription rate with 6 or more items
	Prescription rate of digestive medicines
Medication cost per day of use	Medication cost per day of use
Prescribing expensive medications	Proportion of prescribing of high-priced medicines
	Proportion of cost of high-priced medicines
NSAIDs^a^ and steroids for osteoarthritis	Duplicate prescription rate for NSAIDs
	Prescription rate for steroids

^a^ *NSAIDs* Nonsteroidal anti-inflammatory drugs

Consumers can also see information about PAS results to help them make informed choices when selecting a medical care institution. Since 2006, indicator results, such as the number and the rate of Caesarean sections at each institution, have been openly reported.

## Results

Models of our study on physicians’ perceptions of the PAS, its effects on their behavior, and their responses are summarized in Table 3.

**Table 3:** Physicians’ perceptions and reactions to the PAS and determining factors

Predisposing factors for physician prescribing behavior	Perceptions	Responses
Experience factors		
● Training received during residency periods	● Increased consciousness of their own prescribing behavior	● Initially paying attention to the reports, but beginning to ignore them over time
● Accumulated knowledge through their clinical practice	● Acknowledge the need for appropriate prescription guideline	● Following market trends and/or patient demands instead of cooperating fully with the PAS
Environmental factors		
● Patient demands	● Concerned about violation of their professional autonomy and expertise	● Play smart by up-coding for losses
● Market trends	● Dissatisfied with indicators and ranking methods of the PAS	● Uses non-reimbursable treatment or gives up requesting claims for treatment provided
● Information from seminars or pharmaceutical companies		
Sociopsychological factors		
● Distrust of true intentions and purpose of PAS	● Dissatisfied with methods of notification	
● Distrust of HIRA	● Apprehensive about releasing PAS results to the public	

### Predisposing factors of prescribing behavior

The focus group participants reported that their prescribing behavior was affected both by training received during their residency periods and knowledge accumulated through clinical experience (experiential factors). Their behavior was also affected by patient demands, market trends, and information from seminars or pharmaceutical companies (environmental factors).

#### Clinical autonomy, based on training and accumulated experience

Participants indicated that the most important determining factor was their professional judgment, based on clinical training and accumulated personal experience. They stressed that physicians observe the effects of antibiotics many times in their clinical practice. They emphasized that what they learned during their training is strongly ingrained, so it would be hard for them to be open to other inputs.

“Physicians prescribe antibiotics according to what they learned and what they think works. Physicians have their own background for their decisions.” (G1-a)

“My training was in the respiratory system. As I was taught, I now prescribe inhalants often.” (G1-b)

Participants also argued that diagnosis and prescription may differ for the same symptom or disease, depending on the prescribing physician’s specialty. For example, for symptoms resembling a common cold, prescribing decisions depend on the physician’s specialty—primary care, pediatrics, or otorhinolaryngology—resulting in different diagnoses and treatment.

“Otolaryngologists prescribe antibiotics more often than other specialties. Pediatricians don’t prescribe antibiotics as much. It is because we have many patients who need antibiotics. We have a higher proportion of patients with infections.” (G2-a)

Participants were concerned that the PAS could result in a uniformity that disregards differences between specialties.

#### Patient demands in a competitive market

Regardless of their specialties, participants acknowledged that patient demands were another important factor that affected their prescribing behavior. The participants suggested that, to patients, quick recovery or restoration to health is most important, and this affects patients’ choice of clinics and physicians (‘physician shopping’). Physicians worried that if these expectations were not met, patients could make claims against them. They also asserted that quick recovery is a critical factor in building trust between physicians and patients.

“Maintaining a good relationship with my patients is the most important issue for me. If it is ruined, then nothing works out. I have no other choices.” (G4-a)

Participants admitted that they kept records of patients’ preferences for injections or antibiotics in medical records, and prescribed according to preferences. In particular, geriatric patients were known to prefer injections; therefore these were more frequently provided.

“My injection prescription rate is in the highest range. However, the mean age of my patients is 75 years old. If I refuse to prescribe injections, they get angry. Some cry. Others complain that I don’t respect them. What can I do? Female elderly patients don’t come back if I say ‘no’ to them.” (G4-b)

“Nurses in my clinic told me that I was notorious among patients. When I was in the bottom 20th percentile [in terms of prescribing injections], patients didn’t come back. So I made some changes. Then the number of female elderly patient visits increased. My score now is around the 40th to 50th percentile.” (G4-c)

These opinions seem to contradict the participants’ previous comments that they prescribe according to their own expertise and professionalism. It seems that, in practice, physicians’ expertise and autonomy do not always take precedence over patient demands. Participating physicians stated that prescribing antibiotics is unavoidable because patients choose physicians who will provide their preferred treatment. Physicians’ prescribing behavior is very affected by patient demands, especially when they work in a competitive medical market. Abundant information available through the internet and mass media appears to influence patients, so that they make direct requests for specific medication. Both an otorhinolaryngologist and a primary care physician pointed out that market competition caused difficulties for them. They reported that it is often very tiring and time-consuming to persuade patients asking for antibiotics or injections that this medication is not appropriate for their condition. In a competitive market, adhering to government guidelines makes their work difficult.

“Competition between private clinics is very intense. In the case of GPs, patients don’t wait for us just as they do in university hospitals. If they don’t feel better within one or two days, they just go to see another physician. That’s why GPs tend to prescribe high-priced drugs, antibiotics, injections, or steroids more often in the initial treatment.” (G4-d)

“When I first opened the clinic, I followed what I learned from my school or guidelines from doctors’ associations or HIRA. After one year of practice, the number of patient visits fell to one third. There are so many GPs in my town. If I say injections are not necessary for the patients, they go to another clinic up the road.” (G4-e)

“Mothers insist on branded medicines, even if I prescribe generic drugs. For example, if I prescribe generic drug A to a child, the mother will ask me to prescribe B from company C instead.” (G3-b)

Those who said patient demands played a huge role in their prescribing behaviors argued that patient education is vital to changing prescribing practices. Physicians argued that the government should start a campaign of patient education on the appropriateness of particular medication for various illnesses.

#### Influence of seminars and pharmaceutical companies

Information from seminars, academic conferences, or pharmaceutical companies also influences the prescribing behavior of physicians. Our analysis indicated that physicians’ prescribing behavior is often influenced by pharmaceutical companies’ marketing strategies. Patients often prefer brand name drugs that they have learned about from TV advertisements, while physicians rely on pharmaceutical companies’ marketing materials. In general, participating physicians showed a low level of trust in bioequivalence tests conducted in South Korea between generic and branded drugs.

### Physicians’ perceptions of the PAS

#### Positive perceptions

Regardless of their specialty, participants in all four groups made some positive remarks about the PAS. They reported that the system has been helpful in enabling them to compare their own prescribing behavior with others. Participants also acknowledged the over-use of antibiotics and injections in South Korea. Most of them, including those who had a negative attitude toward the PAS, agreed that changes are necessary. Several participants said that if the program is based on relevant indicators and employs correct data, it has the potential to change physicians’ prescribing behavior.

#### Distrust of the PAS’s intentions and purpose

Overall, participants stressed more negative than positive aspects of the PAS. They described it as another ‘top-down’ managerial intervention by the government. They also had strong suspicions about its purpose. The strongest criticism of the system was whether it was designed to improve the quality of medical services or reduce pharmaceutical expenditure. Most of the participants seemed to assume that its main purpose was to reduce pharmaceutical expenditures, while bolstering the financial security of the National Health Insurance Corporation. Participants therefore expressed antagonistic attitudes toward the use of indicators that measure how often physicians prescribe lower-priced drugs, asserting that these indicators may promote prescription of ‘cheap’ drugs and degrade the quality of medical services.

Many participants said that it is beneficial to be known as someone who prescribes high-priced medicines. They do not want to be known as physicians who are more concerned about cost savings than effective treatment.

“I feel like they are forcing us to prescribe cheap drugs. It is not about improving the quality of medical services but all about drug cost savings. It may help to cut out unnecessarily prescribed drugs, but they excluded all the complex medicines from the positive list. What can I do if I need to prescribe complex medicines for the patient?” (G2-b)

“I am not sure if what they want is appropriate pharmaceutical prescriptions or cost savings.” (G3-a)

#### Deeply rooted distrust of the Health Insurance Review and Assessment Service (HIRA)

Our analysis revealed deeply rooted distrust of the PAS and of HIRA, the agency in charge of the PAS. Study participants reported experiencing unpleasant pressure from, and dissatisfaction with, PAS. Most participants used negative terms such as “threat”, “daunting”, and “pressured” when they described the PAS.

“Auditing intimidates me. I feel I am monitored all the time, which makes me very uncomfortable. I am stressed out. I worry that I may get into trouble.” (G2-c)

“When I received the report from HIRA, it was like receiving a silent threat.” (G4-d)

This well-established distrust seems to come from a lack of effective communication between HIRA and physicians.

“In the end, it is all about partnership. If they are not ready to listen to physicians, it is really a serious problem…whenever we hear that there is a problem, their answer is simple. ‘It’s already been decided.’” (G3-a)

#### Concerns about violation of autonomy and expertise

Similar to the findings of most studies on physicians’ attitudes toward any controlling mechanism [13–15], negative attitudes among physicians in our study appeared to arise from a deeply held belief in clinical autonomy. All participants, regardless of specialty, argued that prescribing decisions should not be controlled by a third party but be based on the expertise of physicians. They claimed to prescribe in line with their medical beliefs as professional physicians, even if this was contrary to guidelines.

“I’m not sure what the word ‘appropriateness’ really means here. I made the decision based on my professional judgment of patients’ conditions and status, so this is a matter between a physician and his/her patient. It is not a matter in which a third party should interfere. The government often thinks that everybody should perform in the same way according to the standardized guidelines, but there are cases that are far from the standard. In my view, only my patient and I can decide what is an appropriate treatment for a specific situation.” (G4-c)

Most of the participants believed that the PAS forces them to prescribe treatment based on rules, and that one result of this single-standard system is a lower quality of medical services.

“The biggest problem with this system is that it may unify all the medical services to a single standard. The system forces us to just follow standardized medical practice guidelines, provided by HIRA, even when they are not consistent with newly published information.” (G1-a)

#### Dissatisfaction with PAS indicators and implementation method

All participants pointed out that, to improve the PAS, valid indicators should be developed and applied. They complained that current indicators do not accurately reflect the quality of medical services. Participants felt that they were evaluated unfairly, based on distorted data. They also argued that the PAS should only check whether physicians meet the criteria or not, rather than ranking them.

“For J06 laryngotracheobronchitis, a steroid should be prescribed. However, from J00 to J06, they fall under the same category of the PAS. Physicians have pointed this out many times but it hasn’t changed yet.” (G3-a)

“Statistics or indicators are all about controlling physicians. Ranking physicians from 1 to 100 does not make sense at all.” (G2-d)

Participants also pointed out that the current system, which allows physicians to register only the main diagnosis when they process claims, gives them an incentive to distort claims.

“[with the current system] there is no way to differentiate between main and sub diagnoses. Some patients may need two or three injections for different diagnoses. When a hypertensive patient came to me because of a nosebleed, I had to register ‘hypertension’ as the diagnosis.” (G2-e)

Most of the participants did not have sufficient understanding of the evaluation indicators and how they are calculated. They expressed vague fears about receiving the PAS results because they had not been clearly informed about the penalties for a low score. This indicates that the purpose, evaluation criteria, and implementation methods of the PAS are not effectively communicated to physicians.

“Is there any penalty for those who get low scores?” (G1-c)

“Many physicians don’t know which one [i.e., medication] is a high-priced drug or what the exact purpose is [of collecting data regarding the] prescribing rates of high-priced drugs.” (G2-b)

Participants also complained that they did not know where to find such information.

#### Dissatisfaction with the notification method and the publication of results

Regardless of their specialty, most of the participants were hostile to the PAS’ use of the term “Notice” and suggested that the title of the “Grade Report” should be changed to a more neutral one such as “Reference” or “Results”. At the same time, they said that publishing results openly to those without the appropriate medical knowledge to properly evaluate the information, is an unwelcome challenge to their medical expertise.

“I don’t think ‘evaluation’ is the right term for this system. The term implies that they investigate who did right and who did wrong. The name should be changed to ‘trend research’ or something like that.” (G1-a)

“The result should not be open to the public. Can you explain to me what an upper or lower respiratory system is? Releasing results to the public, most of whom don’t have medical expertise, can distort the image of the whole healthcare system.” (G3-c)

Participants also had critical opinions of the report format and the evaluation cycle. They preferred graphs and figures to numerical values. Many suggested that evaluations should be conducted once, in the fourth quarter, or perhaps twice a year. For the PAS to be effective in changing physician behavior, they suggested that better feedback methods should be developed. Most physicians do not understand the recommendations which accompany the PAS results. They also thought that there should be appropriate incentives to motivate physicians to change their prescribing behavior. Physicians would be motivated to do so if they could expect positive evaluations and encouragement through rewards such as an excellent clinic certification.

## Discussion

This study was conducted to understand how South Korean physicians perceive the PAS. A total of 27 participants from internal medicine, otorhinolaryngology, pediatrics, and primary care were interviewed to identify factors determining physicians’ prescribing behavior, and the effects of the PAS on this, together with their response to the program. Like previous studies on factors affecting prescribing behavior [16–19], we found that South Korean physicians’ prescribing behavior is determined by both internal and external factors, including their training and experience [20], patient expectations and demands [21], competitive market forces [22], and promotion and marketing [23].

Most of the participants in this study had negative perceptions of the PAS, stemming from a belief that the reports were distorted by inadequate data collection or interpretation. This result is similar to findings in the study of Jones et al. [18], which found that UK physicians seldom use the prescribing analysis and cost data from the Prescription Pricing Authority [24]. However, Axelsson et al., who studied 603 physicians in Sweden, reported that attitudes to the prescribing guidelines were very positive; 42 % of physicians used the guidelines every day, and 34 % every week [25]. Most of the participants in this study acknowledged that excessive antibiotic prescribing is problematic. They all agreed that some changes are necessary, but questioned whether the PAS is the right vehicle. Enforcement by government guidelines does not seem to be an effective way to change physician behavior.

Physicians’ negative perceptions of government policy seemed to come from various sources, including concerns about violations of their autonomy and expertise, suspicion about the program’s intention and purpose, distrust of HIRA, dissatisfaction with notification methods, and concerns about the publication of results. Physicians worried that the current PAS would not only promote conformity to a single standard but would also lower the quality of medical services. Moreover, as a highly autonomous and professional group, participants strongly objected to a system which results in them being evaluated and compared with others in terms of prescriptions written, or rates of injections of antibiotics [26, 27].

Interestingly, we found that physicians’ resistance to perceived violations of their autonomy were often inconsistent and contradictory. While they were very positive about new information or printed materials provided by pharmaceutical representatives, they were less enthusiastic about government guidelines or standards. They appear willing to comply with patient demands as a means of surviving in a competitive market. Physicians’ inclinations to readily accept patient demands in making prescribing decisions has been reported in previous studies [21]. In an interview with a focus group composed of 24 physicians [19], it was found that decisions to prescribe antibiotics were made in the context of the physician–patient relationship to prevent potential tension and patients potentially finding other physicians. This implies that an education campaign for patients may improve the effectiveness of the PAS.

Care is necessary when interpreting physicians’ comments that they had no choice but to comply with patients’ requests for antibiotics, injections, or high-priced drugs. A qualitative study conducted with 8 physicians and 42 patients in North Rhine, Germany, showed that physicians tended to over-assume patient demand for antibiotics [18]. In South Korea, Cho reported that while 73 % of pediatricians thought that patients wanted antibiotics, only 2 % of patients expected them [28]. Petursson conducted qualitative interviews with 16 physicians in Iceland, and found that unstable physician–patient relationships, due to a lack of continuity of care, was the most important reason for prescribing of antibiotics in situations with low pharmacological indications [17]. Although several studies have shown that elderly patients in South Korea prefer injections or antibiotics [29], the claim that physicians had no choice other than to prescribe high-priced drugs, injections, or antibiotics due to patient demand may be somewhat exaggerated.

It is notable that physicians’ negative perceptions of the PAS seemed to be aggravated by suspicion and distrust about the purpose of this program. The most appropriate prescription would be a cost-effective one that provides high quality medical care at low cost. Participants, however, seemed to believe that improvement in the quality of care is only the stated purpose, with cost reduction being the real goal. Physicians expressed a deeply rooted distrust of HIRA, the body in charge of the system. They believed that HIRA’s role is to reduce medication costs by scrutinizing their claims and often rejecting their requests for reimbursements. In fact, the PAS is meant to encourage physicians to improve their practice autonomously, by providing information on their prescribing behavior. PAS is therefore entirely separated from HIRA’s reimbursement review process. Physicians continued to use negative terms such as “threat”, “daunted”, and “pressured” to characterize the PAS, associating the system with their distrust of HIRA.

Since this study was conducted in 2009, there have been several changes in government policy, including P4P (Pay for Performance), which was implemented in 2011; targeted acute myocardial infarction (AMI); and caesarean section delivery. In October 2010, the South Korean government introduced a prescribing incentive scheme to reduce physicians’ prescribing rates, and an online, computerized, prospective drug utilization review (pDUR) has been in operation since December 2010. This seems to have had some effect on overuse and misuse of medicines. Policies that target antibiotic prescription rates for upper respiratory tract infections, overuse of injections, and polypharmacy (the number of medications prescribed ≥6), were implemented in July 2013. Despite this ongoing introduction of new policies, any change in physicians’ prescribing behavior has yet to be shown. Findings from studies that have examined the effectiveness of these policies are not consistent [30, 31]. Despite several interventions since the PAS program started, no research has shown significant effects on prescribing behavior.

## Conclusions

Overall, the results from our study indicate that forming a social consensus on the purpose of the PAS is the most critical prerequisite. Physicians do not agree that they are responsible for cost containment. Participants in our study felt that maintaining the quality of service was the most important issue. They view the PAS as a system that forces physicians to focus on cost rather than quality. Without narrowing such gaps in perceptions and eliminating deeply rooted suspicions, any government efforts will continue to fail.

For HIRA to regain the trust of physicians, it must listen to them more proactively and have open channels for receiving comments and inquiries. Physicians place a higher priority on their professional autonomy and on market competition than on government surveillance. If change is to happen, this cannot be overlooked.
